# PathVLM-Eval: Evaluation of open vision language models in histopathology

**DOI:** 10.1016/j.jpi.2025.100455

**Published:** 2025-06-05

**Authors:** Nauman Ullah Gilal, Rachida Zegour, Khaled Al-Thelaya, Erdener Özer, Marco Agus, Jens Schneider, Sabri Boughorbel

**Affiliations:** a Qatar Computing Research Institute, Hamad Bin Khalifa University, Doha, Qatar; b College of Science and Engineering, Hamad Bin Khalifa University, Doha, Qatar; c Sidra Medicine and Research Center, Doha, Qatar

**Keywords:** VLMs, LLMs benchmarking, Pathology, Zero-shot evaluation

## Abstract

The emerging trend of vision language models (VLMs) has introduced a new paradigm in artificial intelligence (AI). However, their evaluation has predominantly focused on general-purpose datasets, providing a limited understanding of their effectiveness in specialized domains. Medical imaging, particularly digital pathology, could significantly benefit from VLMs for histological interpretation and diagnosis, enabling pathologists to use a complementary tool for faster morecomprehensive reporting and efficient healthcare service. In this work, we are interested in benchmarking VLMs on histopathology image understanding. We present an extensive evaluation of recent VLMs on the PathMMU dataset, a domain-specific benchmark that includes subsets such as PubMed, SocialPath, and EduContent. These datasets feature diverse formats, notably multiple-choice questions (MCQs), designed to aid pathologists in diagnostic reasoning and support professional development initiatives in histopathology. Utilizing VLMEvalKit, a widely used open-source evaluation framework—we bring publicly available pathology datasets under a single evaluation umbrella, ensuring unbiased and contamination-free assessments of model performance. Our study conducts extensive zero-shot evaluations of more than 60 state-of-the-art VLMs, including LLaVA, Qwen-VL, Qwen2-VL, InternVL, Phi3, Llama3, MOLMO, and XComposer series, significantly expanding the range of evaluated models compared to prior literature. Among the tested models, Qwen2-VL-72B-Instruct achieved superior performance with an average score of 63.97% outperforming other models across all PathMMU subsets. We conclude that this extensive evaluation will serve as a valuable resource, fostering the development of next-generation VLMs for analyzing digital pathology images. Additionally, we have released the complete evaluation results on our leaderboard PathVLM-Eval: https://huggingface.co/spaces/gilalnauman/PathVLMs.

## Introduction

In recent years, large language models (LLMS)^2^^,^^7^^,^^17^^,^^47^^,^^48^^,^^54^^,^^55^^,^^69^ have achieved remarkable advances in their ability to understand, reason, and generate textual content for a wide range of open-ended tasks. Utilizing the foundational generality of LLMs, VLMs^1^^,^^16^^,^^20^^,^^35^^,^^37^^,^^46^^,^^53^^,^^56^ have further demonstrated state-of-the-art capabilities in handling and understanding multiple modalities, such as text, images, audios, and videos. This visual and textual integration making them highly applicable to complex and unconstrained scenarios. However, despite the extensive capabilities of these foundation models, existing benchmarks^19^^,^^62^^,^^64^ cater predominantly to general domain applications. This limited scope fails to capture the full range of challenges and opportunities presented by domain-specific contexts, particularly in critical fields like histopathology. Histopathological analysis plays a vital role in disease diagnosis and treatment planning in large-scale clinical applications. However, traditional pathological processes face significant challenges, as they rely heavily on specialized expertise and labor-intensive manual examination, often resulting in diagnostic delays and variability. These challenges underscore the need for emerging methods of artificial intelligence (AI) in histopathology. VLMs have shown promising capabilities to address these limitations by simultaneously processing and analyzing histological image data and associated clinical information.^26^^,^^52^

Given the critical demands for precise interpretation in the field of histopathology, conducting comprehensive evaluations of LMMs’ abilities to interpret digital pathology images is essential. However, the field still faces a notable scarcity of high-quality benchmark datasets.^52^ Currently, only two public datasets are available for pathology: PathVQA,^26^ designed for visual question-answering tasks in pathology, and the extensive PathMMU dataset, which is formatted into multiple choice questions (MCQs). PathMMU introduced the first pathological-focused dataset designed for the evaluation of VLMs, which included MCQs derived from real-world pathology images and clinical scenarios.^52^ The contribution of PathMMU laid the foundations for AI-driven pathology research by highlighting the potential of VLMs to assist diagnostics. However, its scope was limited to a narrow range of models, leaving a need for more extensive benchmarking across the rapidly evolving landscape of larger and more advanced models. The benchmarking pipeline in PathMMU is not flexible to easily evaluate the large resource of publicly available models on Hugging Face (HF) platform. To address this limitation, our study builds upon PathMMU by leveraging the VLMEvalKit framework^19^ to evaluate over more than 60 state-of-the-art visual language models (VLMs) on histopathology image datasets. The chosen pool of models covers a wide spectrum of architectures and sizes, allowing users to make decisions which model is suited for their use case. By introducing a diverse range of histopathology-focused queries and evaluating models across various conditions, we aim to answer critical questions such as: Can VLMs effectively interpret histopathology data without clear visual cues? Do larger models outperform smaller ones in complex histopathology diagnostic workflow? And how much do vision components contribute to the diagnostic accuracy of these models? Which model family scales better? Do existing benchmarking datasets exhibit a strong dependence between text and visual information in the questions? Our work aims to provide clarity on these questions, offering insights that could guide future advancements in AI-driven histopathology diagnostics.

We present the first large-scale benchmarking of VLMs in pathology using the VLMEvalKit framework.^19^ We aim to extend existing efforts by evaluating a broader range of models and providing a more detailed understanding of how model size and architecture influence performance on pathology tasks. In addition to using dataset like PathMMU, we incorporate the LMMs-Eval methodology^64^ to ensure a comprehensive evaluation that captures both the visual and textual capabilities of these models.

Our contributions in this article are as follows:

• **Extensive benchmarking:** We present the most comprehensive evaluation of VLMs on pathology image datasets to date, covering more than 60 models using the VLMEvalKit framework.^19^
• **Model selection insights:** We provide insights into the performance of various models, highlighting the relationship between model size and performance in histopathology diagnostics.
• **Evaluation across visual conditions:** We evaluate models under different visual conditions (Original Image, Blackout, Fully Blurred, and Partially Blurred) to understand their reliance on visual inputs. This evaluation sheds light on the ability of VLMs to process textual information when visual cues are degraded or absent. This also highlights a limitation in current histopathology benchmarking datasets which requires future work to increase the dependency textual questions and images.
• Public Leaderboard: Our leaderboard is now publicly available at https://huggingface.co/spaces/gilalnauman/PathVLMs, enabling future comparisons and promoting research in histopathology-focused VLMs. It is regularly updated as new models are evaluated.

## Related work

The recent progress in VLMs^1^^,^^12^^,^^19^^,^^20^^,^^32^^,^^52^^,^^54^^,^^59^^,^^64^ has driven advancements in AI, enabling machines to learn, interpret, and reason across multiple modalities such as text and images. This section provides an overview of the recent open-resource VLMs, evaluation methodologies, and benchmark datasets for general-purpose,^29^^,^^30^^,^^38^^,^^42^^,^^59^, ^60^, ^61^ and medical^6^^,^^26^^,^^28^^,^^52^^,^^67^ that have shaped the development and evaluation of VLMs.

### Large multimodal models

Over the past few years, AI has undergone a transformative evolution, largely driven by the introduction of transformers in language models^55^ and Vision Transformers (ViT).^4^ Language models like BERT,^17^ GPT-3,^7^ and T5^51^ laid the foundation for the unprecedented success of LLMs. Subsequent iterations, including LLaMa,^54^ ChatGPT,^47^ GPT-4,^2^ and Vicuna,^13^ have set benchmarks in natural language understanding, context-aware text generation, and complex reasoning tasks. Simultaneously, ViT^4^ revolutionized computer vision by introducing attention-based models capable of learning global dependencies in image data. This convergence of natural language and vision models has catalyzed the development of VLMs, enabling seamless integration of diverse modalities.

Prominent VLMs such as CLIP,^50^ Flamingo,^3^ and BLIP-2^31^ have demonstrated robust multimodal reasoning and integration capabilities. These foundational works inspired a wave of advanced VLMs, including GPT-4V,^46^ Gemini Pro Vision,^53^ LLaVA,^37^ LLaVA-NeXT,^35^ Qwen-VL,^5^ Qwen2-VL,^56^ MiniGPT-4,^71^ InstructBLIP,^15^ InternVL,^12^ XComposer,^65^ Phi3,^1^ Molmo,^16^ and LLama3.^20^ These models have achieved significant milestones in tasks such as visual question answering (VQA),^33^^,^^63^^,^^67^ OCR capabilities,^39^ document understanding,^44^^,^^49^ and much more. In this work, we focus on medical type of data, specially MCQs in pathology.^26^^,^^52^

Despite this progress, most VLMs have been developed and evaluated on general-purpose datasets, leaving domain-specific applications, particularly in medical pathology, underexplored. In this work, we address this gap by focusing on the unique challenges presented by pathology data, specifically MCQs in the PathMMU dataset.^52^ Our study benchmarks over 60 state-of-the-art VLMs on this dataset, representing the most comprehensive evaluation to date. This work sets the stage for future innovations in multimodal learning for critical applications in pathology.

### Benchmarks for VLMs

With the rapid advancement in VLMs, the development of architectures, and datasets has flourished. However, evaluating these models remains a critical aspect of understanding their effectiveness and limitations. Benchmarks plays an important role by providing a standardized framework for assessing model performance across diverse tasks and datasets. VLMs cover a wide range of benchmark datasets, including both general and domain-specific benchmarks.

#### General domain benchmarks

General domain benchmarks focus on evaluating the fundamental perception abilities of LLMs. Notableexamples inlcude LAMM,^60^ LVLM-eHub,^59^ MM-Vet,^61^ SEED,^30^ MMBench,^38^ MathVista,^42^ and BenchLMM,^8^ which have been used to assess the basic perception abilities of large models. Recently, more advanced benchmarks have emerged, such as MMMU,^62^ MME-RealWorld,^68^ UNK-VQA,^24^ SEED-Bench,^29^ LMMs-Eval,^64^ VLM-EVAL-Kit,^19^ and benchmarks for cultural understanding.^45^

#### Medical specific benchmarks

In the medical domain, specialized benchmarks evaluate the capabilities of VLMs in complex diagnostic tasks: VQA-RAD^28^ and VQA-Med^6^ offer collections of Q&A pairs derived from radiology images, facilitating evaluations of radiological diagnostic capabilities. Additionally, PMC-VQA^67^ is a large-scale dataset that generates numerous Q&A pairs by prompting ChatGPT with text-only image captions. Despite the progress in medical VLMs, the application of these models in specific sub-fields, particularly in histopathology remains underexplored due to the lack of high-quality benchmarking datasets. In the public domain, only two major datasets are available for pathology:

• PathVQA^26^: A dataset designed for VQA in pathology, featuring over 30,000 samples of 4998 images. However, PathVQA has limitations, as its samples are derived from a narrow source (textbooks), and the use of heuristic-based caption-to-question conversion often leads to less diverse and less logically coherent Q&A pairs.
• PathMMU^52^: A massive and comprehensive dataset tailored for pathology, featuring MCQs and diverse task formats. PathMMU represents a significant step forward in providing a high-quality resource for evaluating VLMs in the field.

The scarcity of histopathology-specific benchmarks highlights the need for more diverse and logically curated datasets to better evaluate VLMs’ capabilities in specialized tasks. Our study addresses this gap by conducting extensive evaluations of VLMs on the PathMMU dataset, providing actionable insights for advancing AI in histopathology. Therefore, in this study, we address the gap in histopathology-specific benchmarks by conducting an extensive evaluation of VLMs using VLM-Eval-kit framework^19^ on the PathMMU^52^ dataset.

## PathVLM-Eval

In this section, we provide an overview of recent open-source VLMs and available datasets for general-purpose and medical domains to evaluate VLMs. We exclude commercial models from our benchmarking as a design choice to focus on open-source VLMs, ensuring transparency, reproducibility, and accessibility for the research community. Additionally, because we plan to maintain an up-to-date leaderboard, including commercial models would be cost-prohibitive.

### Models

This study provides a comprehensive evaluation of over 60 open-source VLMs using the VLMEvalKit framework.^19^ Building upon this framework, we have developed a large-scale benchmarking approach, referred to as the PathVLM-Eval toolkit. PathVLM-Eval enables an in-depth analysis of the diverse and rapidly evolving landscape of VLMs.

The evaluation encompasses both general and medical domains, with a particular emphasis on pathological analysis tasks, grouped by main architectural families. The LLaVA series demonstrating significant architectural diversity. These models range from 7 billion (7B) to 34 billion (34B) parameters, including versions such as LLaVA-v1.5 (7B/13B).^58^ These diverse architectures seamlessly integrate vision and language capabilities, supporting a wide range of tasks through various parameter scales, contributing to advancements in multimodal model design. Furthermore, the LLaVA-Next-Interleave models (7B and 7B-DPO)^36^ represent notable advancements within the LLaVA family, particularly with the integration of visual models. The study also incorporated specialized implementations of next-generation variants such as LLaVA-Next-Vicuna (7B and 13B), LLaVA-Next-Mistral (7B), LLaVA-Next-Llama3, and LLaVA-Next-Interleave models (7B and 7B-DPO),^36^ LLaVA-along with additional architectures like InternLM-7B, XTuner-optimized versions (LLaVA-v1.5-7B-xtuner and LLaVA-v1.5-13B-xtuner), and LLaVA-LLaMA-3-8B.^14^ This diverse array of architectures showcases LLaVA’s evolution in vision-language integration, demonstrating how different model configurations and parameter scales contribute to advancing the multimodal large language systems.

In addition to the LLaVA family, we also evaluated the ShareGPT4V and Qwen VL families, both demonstrate significant advancements in vision–language integration. The ShareGPT4V family includes models such as ShareGPT4V-7B and ShareGPT4V-13B,^9^ which have shown notable improvements in multimodal learning. The Qwen VL family encompasses a wide range of model sizes, from 2B to 72B parameters, with multiple instruction tuned configurations. This family includes base model such as Qwen-VL.^5^ along with instruction tuned variants and their quantized versions such as Qwen2-VL-7B, Qwen2-VL-7B-Instruct-GPTQ-Int4, Qwen2-VL-7B-Instruct-GPTQ-Int8, Qwen2-VL-2B-Instruct, Qwen2-VL-2B-Instruct-GPTQ-Int4, Qwen2-VL-2B-Instruct-GPTQ-Int8, and the large-scale Qwen2-VL-72B.^57^ The Qwen family advances VLMs by improving instruction following and vision- language understanding.

Other notable families of emerging architectures include Monkey,^32^ Monkey-Chat,^40^ and MiniMonkey,^27^ as well as the Phi-3-Vision and Phi-3.5-Vision.^1^ Furthermore, the InternVL variants^10^, ^11^, ^12^^,^^23^, ShareCaptioner,^9^ and the InternLM-XComposer2 series, including InternLM-XComposer2-1.8B and InternLM-XComposer2,^18^^,^^66^ further expand the landscape of multimodal systems. Specialized model collections, such as MolmoE-1B, Molmo-7B-D,Molmo-7B O,^1^ XinYuan-VL-2B-Instruct,^2^ MolmoE-1B, Molmo-7B-D,Molmo-7B-O,^3^ MMAlaya,^41^ Llama-3.2-11B-Vision-Instruct,^4^ and Ovis variants^43^ have significantly enhanced our benchmarking framework. These models offer a thorough representation of the capabilities of contemporary VLMs. The computational architectures evaluated ranged from 1 billion to 72 billion parameters, utilizing VLMEvalKit framework on an extensive PathMMU pathology dataset.

### Dataset

In this study, we focus on the histopathological dataset PathMMU^52^ a large-scale pathology benchmark designed to evaluate VLMs in domain-specific reasoning and understanding. It consists of five primary subsets:

1. PubMed: A collection of pathology-related MCQs derived from PubMed literature.
2. SocialPath: Pathology knowledge sourced from Twitter.
3. EduContent: Pathology MCQs curated from structured educational content, including textbooks and courses.
4. PathCLS: A set of pathology classification tasks requiring models to identify specific pathological features.
5. Atlas: A dataset derived from histopathology image atlases, involving complex diagnostic reasoning.

In this study, we focus on three key subsets, PubMed, SocialPath, and EduContent due to accessibility and ease of standardization. The PathCLS and Atlas subsets were not included in our evaluations due to data acquisition constraints and their specialized nature. To provide a clear understanding of how PathMMU is structured, Fig. 2 illustrates a sample from the dataset following a VQA format.

## Benchmarking results

In this study, we evaluated over 60+ recent VLMs on the PathMMU dataset, which features diverse tasks within pathology. This benchmark builds upon prior work by Sun et al.,^52^ significantly expanding the evaluation scope by incorporating extensive visual and multimodal conditions using VLMEvalKit.^19^ We summarize the benchmarking results of VLMs on PathMMU in Table 1. For complete results, visit our benchmarking leaderboard PathVLM-Eval: https://huggingface.co/spaces/gilalnauman/PathVLMs or refer to the supplementary material (Section S1, Table S1).

**Table 1:** Evaluation results of VLMs on selected benchmarks in pathology: We benchmarked over 60 models on the PathMMU^52^ dataset using the VLMEvalKit^19^ framework. In Table 1, we show more representative and recent open-source VLMs (till January 01, 2025). We calculated the average score and ‘Params (B)’ represented the total number of billion parameters.

VLM name	Size (B)	Language model	Vision model	Avg score	PubMed		SocialPath		Edu Content	
					Tiny (281)	All (3068)	Tiny (229)	All (1805)	Tiny (255)	All (1938)
Qwen2-VL-72B-Instruct	∼70	Qwen2-72B	QwenViT	**64.0**	75.1	61.0	68.6	55.4	68.2	55.6
InternVL2.5-78B	∼70	Qwen-2.5-72B	InternViT-6B-v2.5	62.5	71.2	57.3	67.2	54.6	71.8	52.8
InternVL2.5-38B	28-40	Qwen-2.5-32B	InternViT-6B-v2.5	**62.7**	65.8	61.4	62.9	57.3	69.4	59.5
InternVL2-40B	28-40	Nous-Hermes-2-Yi-34B	InternViT-6B	56.3	64.0	52.7	59.4	48.9	64.3	48.2
Ovis1.6-Gemma2-27B	28-40	Gemma2-27B	SigLIP-400M	54.7	61.9	49.9	57.2	50.4	58.4	50.6
Qwen2-VL-7B	7-14	Qwen2-7B	ViT-600M	**55.4**	62.6	54.8	57.2	49.9	56.9	50.8
Ovis1.5-Llama3-8B	7-14	Llama-3-8B-Instruct	SigLIP-400M	53.0	61.6	48.7	59.4	49.4	52.9	46.0
InternVL2-8B	7-14	InternLM2.5-7B	InternViT-300M	49.9	58.0	45.3	57.6	44.0	51.8	42.7
Molmo-7B-D-0924	7-14	Qwen2-7B	CLIP ViT-L/14	42.3	47.3	41.3	43.2	41.6	41.2	39.3
LLaVA-Next-Llama3	7-14	Llama-3-8B-Instruct	CLIP ViT-L/14	41.5	47.7	40.4	43.7	38.0	42.4	36.7
LLaVA-v1.5-13B	7-14	Vicuna-v1.5-13B	CLIP ViT-L/14	39.3	45.9	37.3	39.7	37.4	39.6	35.6
Molmo-7B-O-0924	7-14	Qwen2-7B	CLIP ViT-L/14	38.9	46.3	38.1	39.3	36.5	37.6	35.5
MolmoE-1B-0924	7-14	OLMoE-1B-7B-0924	CLIP ViT-L/14	37.4	39.5	36.9	40.6	37.0	35.3	35.1
LLaVA-v1.5-7B	7-14	Vicuna-v1.5-7B	CLIP ViT-L/14	37.4	43.8	39.6	36.2	38.0	31.4	35.3
Qwen-VL-Chat	7-14	Qwen-7B	ViT-G/16	34.5	39.1	31.4	35.4	32.2	37.2	31.7
Qwen-VL-7B	7-14	Qwen-7B	ViT-G/16	32.4	32.4	32.3	30.6	31.9	36.1	31.5
Mini-InternVL-Chat-4B-V1.5	<4	Phi-3	InternViT-300M	**52.3**	55.5	50.7	57.2	46.6	55.7	48.2
InternVL2-4B	<4	Phi-3	InternViT-300M	49.2	57.6	45.5	55.5	42.9	50.2	43.7
Phi-3.5-Vision	<4	Phi-3.5	CLIP ViT-L/14	43.8	49.1	44.2	45.0	40.6	43.9	39.9
Phi-3-Vision	<4	Phi-3	CLIP ViT-L/14	39.0	44.8	38.6	38.9	38.8	36.1	37.1

### Comparison with state-of-the-art benchmarking frameworks on histopathology

We conducted a comprehensive comparison of VLMs evaluated on PathMMU across three benchmarking frameworks: PathMMU,^52^ LMMs-Eval,^64^ and VLMEvalKit.^19^ Table 2 represents a comparison of VLMs that have been evaluated across all three benchmarking frameworks: PathMMU,^52^ LMMs-Eval,^64^ and VLMEvalKit.^19^ The selected models are consistently available in all frameworks, ensuring a fair comparison of performance across different evaluation methodologies. This provides insights into the impact of evaluation strategies and dataset contamination handling in pathology-related multimodal model benchmarking.

**Table 2:** Comparision of VLMs: We compare the results with existing benchmarks from PathMMU^52^ to assess performance using LMMs-Eval,^64^ VLMEvalKit^19^ frameworks.

Frameworks	Model	PubMed		SocialPath		EduContent	
		Tiny (281)	All (3068)	Tiny (229)	All (1805)	Tiny (255)	All (1938)
PathMMU^52^	LLaVA-1.5-7B	41.6	39.9	37.9	38.1	32.5	36.5
LMMs-Eval^64^	LLaVA-1.5-7B	45.5	38.1	34.50	35.79	34.5	35.0
VLMEvalKit^19^	LLAVA-1.5-7B	43.77	39.6	36.34	38.01	31.37	35.34
PathMMU^52^	LLaVA-1.5-13B	44.5	41.0	40.4	40.4	34.1	39.4
LMMs-Eval^64^	LLaVA-1.5-13B	47.3	40.8	42.79	41.50	36.47	38.75
VLMEvalKit^19^	LLAVA-1.5-13B	45.90	37.32	39.73	37.45	39.60	35.60
LMMs-Eval^64^	LLaVA-1.6-Vicuna-7B	41.2	37.1	35.50	38.17	37.65	37.20
VLMEvalKit^19^	LLaVA-NeXT-Vicuna-7b	43.06	36.34	39.30	34.63	35.29	34.52
LMMs-Eval^64^	LLaVA-1.6-Vicuna-13B	39.15	33.08	31.0	32.96	27.45	27.86
VLMEvalKit^19^	LLaVA-NeXT-Vicuna-13b	40.93	37.71	42.36	36.45	34.90	33.95
LMMs-Eval^64^	LLaVA-1.6-Mistral-7B	6.41	5.61	4.37	5.60	4.71	5.52
VLMEvalKit^19^	LLaVA-NeXT-Mistral-7b	47.33	36.83	39.30	34.85	40.39	34.78
PathMMU^52^	Qwen-VL-7B	34.9	32.9	33.6	35.8	34.1	33.6
LMMs-Eval^64^	Qwen-VL-7B	35.23	35.14	35.81	35.96	40.0	37.7
VLMEvalKit^19^	Qwen-VL-7B	32.38	32.26	30.56	31.91	36.07	31.52

### Quantized models in VLMs

In this section, we evaluate quantized variants of VLMs on the PathMMU dataset using VLMEvalKit,^19^ comparing their performance against full-precision counterparts. Table 3 summarizes the results, demonstrating how quantization affects model accuracy across the PubMed, SocialPath, and EduContent subsets. Key observations from quantized VLMs:

**Table 3:** Quantized VLMs on pathology: Quantized models on PathMMU dataset using VLMEvalKit^19^ framework.

Model	Avg. score	PubMed		SocialPath		EduContent	
		Tiny (281)	All (3068)	Tiny (229)	All (1805)	Tiny (255)	All (1938)
Qwen2-VL-7B	55.4	62.6	54.8	57.2	49.9	56.9	50.8
Qwen2-VL-7B-Instruct-GPTQ-Int4	56.15	62.27	53.22	55.45	49.97	65.07	50.92
Qwen2-VL-7B-Instruct-GPTQ-Int8	55.57	62.98	54.62	58.07	49,97	56.86	50.92

***Minimal degradation in performance.*** Qwen2-VL-7B-Instruct-GPTQ-Int4 exhibits only a slight reduction in accuracy compared to the full-precision Qwen2-VL-7B, making it a strong alternative for memory-efficient deployments, whereas GPTQ-Int8 performs comparably to the full model while providing computational savings.

***Trade-offs between speed and accuracy.*** Lower-precision models (GPTQ-Int4) achieve faster inference speeds while sacrificing minimal accuracy, making them ideal for real-time applications. Higher-precision quantization (GPTQ-Int8) retains more accuracy but has moderate computational savings compared to full-precision models.

***Potential for domain-specific optimization.*** Despite quantization, Qwen2-VL models retain strong generalization ability across the PathMMU pathology benchmarks. Future improvements may explore fine-tuning of quantized models on domain-specific medical datasets to further enhance performance. Quantized models offer a viable path toward deploying high-performing VLMs in clinical and real-world applications where computational efficiency is critical. Our benchmarking results indicate that quantization can significantly reduce resource requirements with minimal accuracy trade-offs, paving the way for scalable, cost-effective AI solutions in pathology.

## Analysis

In this section, we provide an analysis on VLMs by different visual conditions, model size vs performance gain, and the correlation between general-purpose VLMs and pathological using VLMEvalKit.^19^

### Contribution visual in VLMs in histopathology

To assess the impact of visual information on the performance of VLMs in pathology-related tasks, we conducted a series of experiments using different datasets: PubMed, SocialPath, and EduContent. These datasets were evaluated under various image conditions to understand how image clarity and visual features influence the model’s ability to answer pathology-related MCQs. The following visual conditions were applied (Fig. 3):

• Original image: High-quality images providing full visual information.
• Blackout condition: Images rendered entirely black, effectively removing any visual input.
• Fully blurred condition: Images blurred to a high extent, removing most of the visual details.
• Partially blurred condition: Images blurred to a lesser extent, preserving some recognizable features.

The results of these experiments are illustrated in Fig. 4, which shows barplots comparing the accuracy of three models across the different visual conditions and datasets. The models evaluated on Qwen2-VL-72B-Instruct. The following key observations were made, one of the most striking observations from the evaluation is that even when the images are completely blacked out (i.e., no visual information is provided), the models were still able to answer a significant portion of the questions correctly. This suggests that many of the questions across these datasets can be answered based on textual information alone. However, it is important to note that there was still a performance drop in the blackout condition compared to the original image condition, particularly in datasets like PubMed and SocialPath, indicating that visual cues do provide valuable context for some questions. The partially blurred condition often retained better performance compared to the fully blurred and blackout conditions. This implies that even partially degraded images can still provide valuable context for the models to interpret the questions correctly. The fully blurred condition, which removes most of the visual details, resulted in more performance degradation compared to the partially blurred condition. This highlights the importance of image clarity in visual tasks. Our analysis reveals that the complete removal of visual information (blackout condition) does not entirely inhibit the models’ ability to answer questions. This is an important insight, as it shows that some pathology-related MCQs can be answered without any visual input. This could be due to the textual cues in the questions and answers that provide sufficient context for the models to make accurate predictions.

This observation aligns with the concept that some pathology-related questions are inherently text-driven and do not require visual input. For example, questions that ask about general medical knowledge or describe common pathological patterns can often be answered without seeing the image. In contrast, questions that require the recognition of histological patterns, cell shapes, or staining characteristics are more likely to depend on visual input.

### Model size vs performance analysis

The relationship between model size and performance is a critical aspect in evaluating the capabilities of VLMs. To gain insights, we analyze the performance of models across different sizes, categorized into three clusters based on the number of parameters:

• Small models (≤10B): These models are lightweight and optimized for resource-constrained settings or on-the-go applications and gain reasonable performance, include *MiniMonkey,*^27^ *Phi3,*^1^ and smaller variants of the *LLaVA*^34^ and *InternVL* families.^10^, ^11^, ^12^^,^^23^ While cost-effective, their performance is typically clustered in the lower to middle range.
• Moderate models (10B–30B): These models offer a balanced trade-off between computational efficiency and performance, making them suitable for versatile real-world applications. Examples include models such as *InternVL*, *Ovis*, and *Phi3*. Models in this range often compete closely with larger models and demonstrate versatility across domains.
• Large models (>30B): These high-performing models such as *Qwen2*, *InternVL2.5*, and *Ovis* dominate this group. They excel in generalization and domain adaptability, but come with significant computational demands.

Given the fact that, whereas larger models often achieve higher performance, the relationship is not strictly linear. Smaller well-optimized models, such as *MiniMonkey*, achieve competitive performance compared to models of moderate size. Examining family-specific trends, the *Qwen2* family consistently leads in the large-model cluster, showcasing effective scalability and optimization. The *InternVL* family spans all three clusters, demonstrating its architectural versatility. The finding reveals inherent trade-offs in model selection, smaller models are resource-efficient but struggle with complex tasks; moderate models offer the best trade-off between performance and cost; whereas large models deliver superior performance at a higher computational expense.

**Fig. 1:**
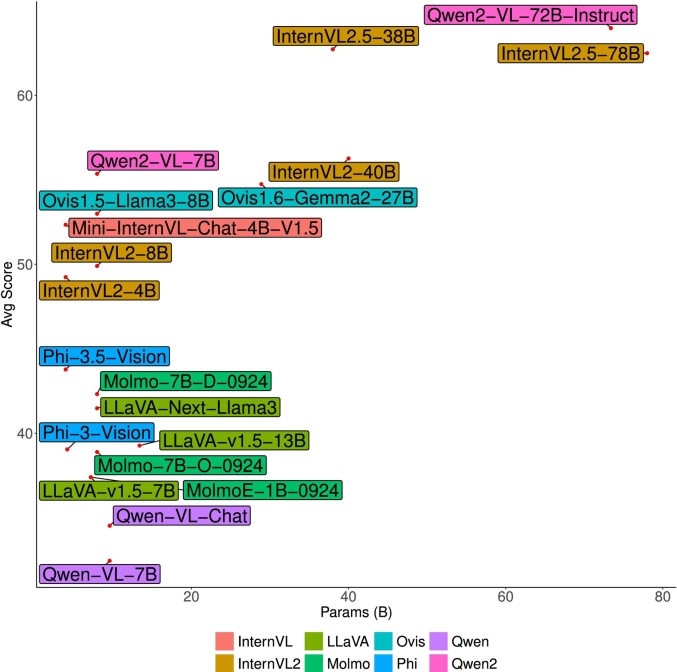
Benchmark summary of representative models from our analysis. The x-axis depicts model size in billion parameters and the y-axis indicates the average performance across the PathMMU’s subsets (Pubmed, SocialPath, and EduContent) datasets we used for evaluation.

Furthermore, visual representation (see in Fig. 1) illustrates the relationship between model size (in billions of parameters) vs average performance highlights the clustering of models and their relative performance across datasets.

**Fig. 2:**
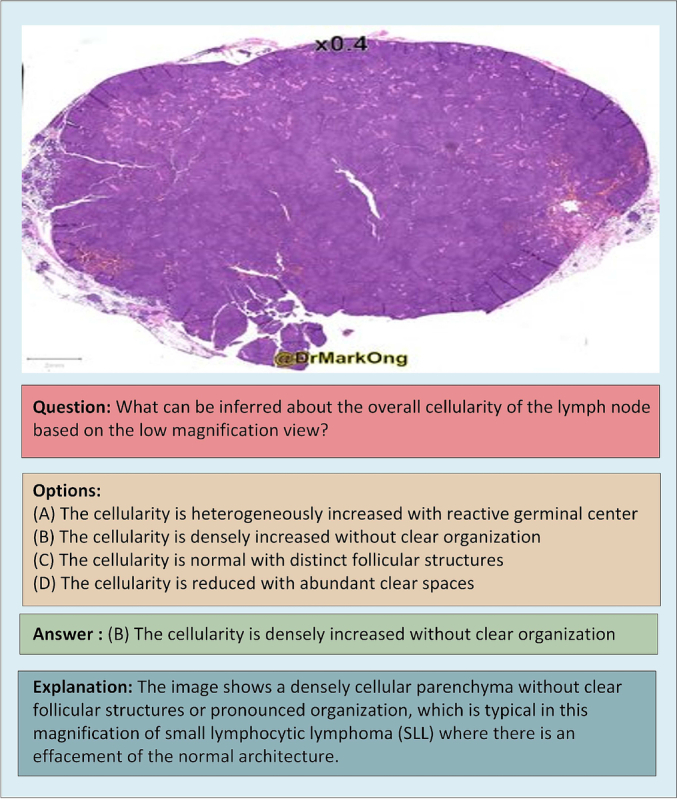
PathMMU dataset format. A sample from the PathMMU dataset contains: a histopathology slide; a clinical relevant question; possible Options (A, B, C, and D); answer (the ground-truth label); and a brief explanation for the correct answer.

**Fig. 3:**
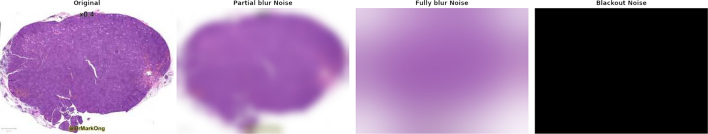
Different types of visual condition (Noise): Illustration of different visual conditions (noise type) used to evaluate the VLMs: original image (full visual information); partially blurred condition (moderate degradation of visual details); fully blurred condition (highly degraded visual details); and blackout condition (zero-vision).

**Fig. 4:**
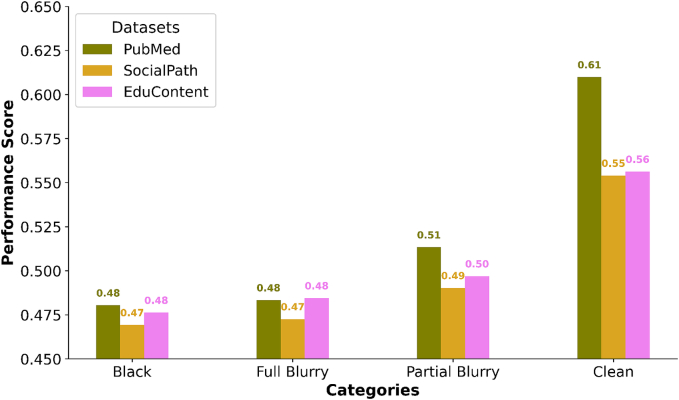
Performance analysis across different visual conditions. Performance of Qwen2-VL-72B-instruct across the PathMMU three subsets: PubMed, SocialPath, and EduContent.

**Fig. 5:**
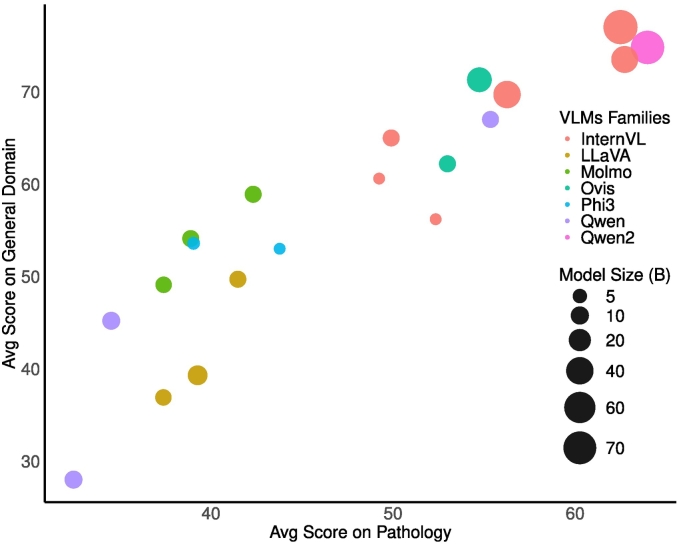
PathMMU vs general dataset performance: Comparison of performance on PathMMU (medical datasets) and general datasets, with bubble size indicating model size (in billions of parameters) and colors representing families. Larger models generally perform better across both domains, with notable contributions from Qwen2VL^57^ and InternVL families.^10^, ^11^, ^12^^,^^23^

**Fig. 6:**
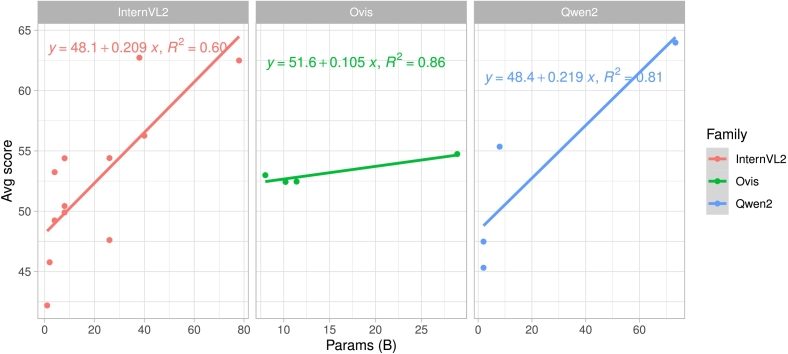
Scaling behavior of vision-language models in histopathology. Illustration of how model size (in billions of parameters) influences average performance across three major VLM families: InternVL2 (red), Ovis (green), and Qwen2 (blue). Qwen2 models exhibit the most stable and predictable performance scaling, whereas InternVL2 models show higher variance, suggesting that factors beyond size impact performance.

**Fig. 7:**
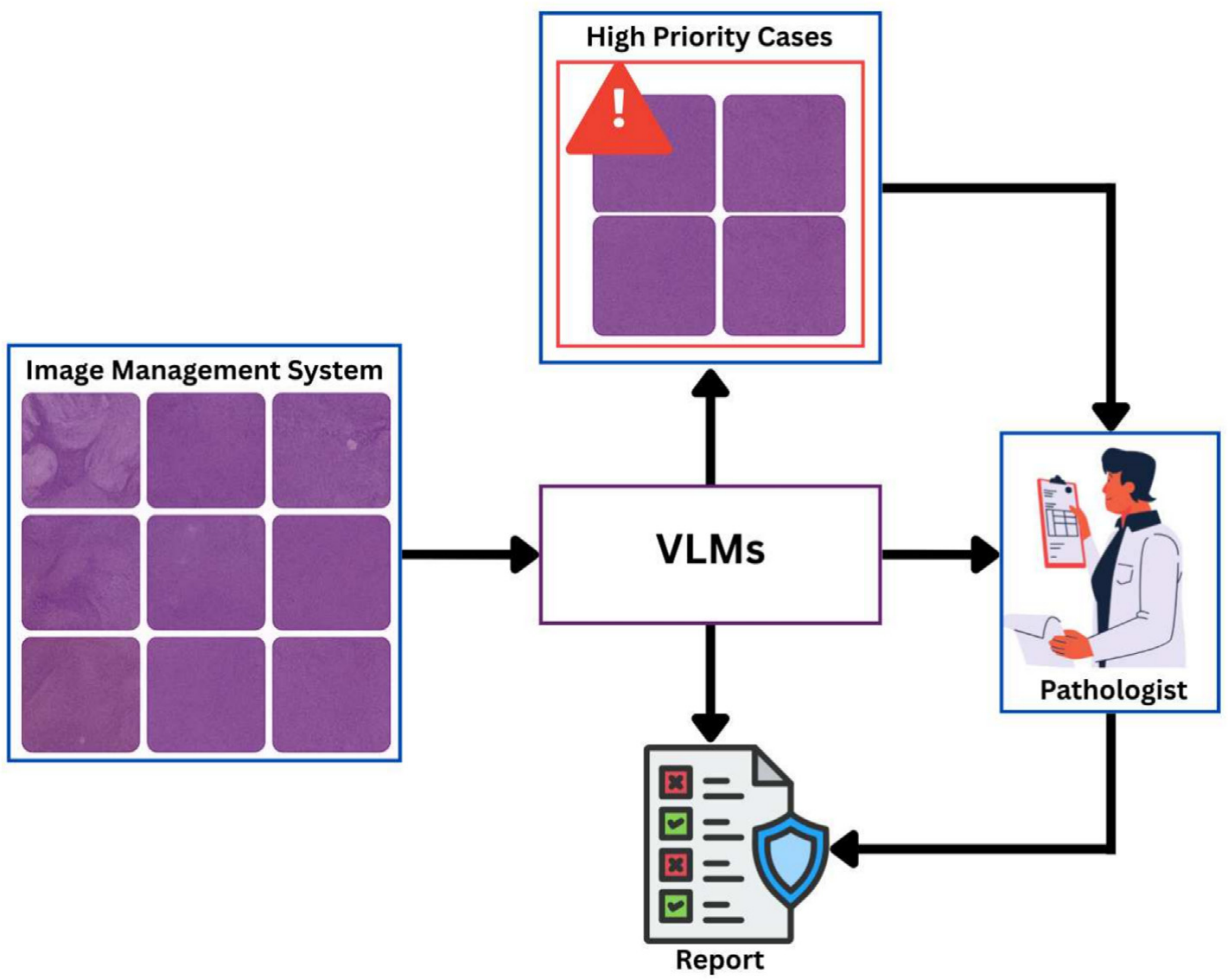
Workflow for integrating VLMs into clinical digital pathology. The whole slide image in the image management system are screened by the VLM. High-priority cases, e.g., cancerous, are identified and flagged for early review by the pathologist. The VLM also pre-fills the case report with general and diagnostic information. The pathologist independently reviews the case and corrects and approves the suggested edits to generate a final report. These VLM applications illustrate two potential in the clinical workflow: Model-based triaging and human expert-in-the-loop case reporting.

### General benchmarks vs pathology and model ranking

Fig. 5 highlights the relationship between model performance on PathMMU (medical datasets) and general datasets. The analysis reveals several critical insights into how VLMs perform across domains, emphasizing key patterns, including trade-offs between performance, computational resources, and domain-specific adaptability.

#### Cross-domain correlation

• A positive correlation exists between performance on PathMMU and general datasets, suggesting that models excelling at general tasks often extend their capabilities to domain-specific pathology tasks.
• High-performing models, such as Qwen2-VL-72B and InternVL2.5-78B, consistently achieve superior results across both domains, showcasing their adaptability and effective cross-domain tuning.

#### Family-specific trends

• **Qwen2 family:** Consistently dominates both PathMMU and general datasets. Demonstrates the effectiveness of scalable architectures and robust optimization strategies, particularly in large-model clusters.
• **InternVL family:** Spans a wide range of parameter sizes, from small to very large models. Highlighting architectural versatility, offering scalable solutions for varied computational and domain-specific requirements.
• **Phi-3 family:** Performs strongly on PathMMU, indicating a focus on domain-specific tasks like pathology. Slightly lags in general datasets, underscoring the trade-offs of specialization.
• **LLaVA family:** Shows progressive improvements in the “Next” series, reflecting ongoing enhancements in architecture and training approaches for better performance.

Furthermore, smaller families such as Ovis and MiniMonkey, deliver competitive performance, particularly on PathMMU, by emphasizing architectural efficiency and targeted optimizations.

### Scaling trends of VLMs in pathology

The relationship between model size (in billions of parameters) and average performance score on the PathMMU dataset is analyzed for three major VLM families: InternVL2, Ovis, and Qwen2. Fig. 6 presents a comparative study with linear regression fits for each model family, demonstrating the performance scaling behavior.

From Fig. 6, on the right side, InternVL2 family (Red), exhibits a moderate positive correlation between model size and performance. The slope of the regression line **(0.209)** suggests significant performance variability, indicating that factors beyond model size (e.g., training data, architecture) influence results. From Fig. 6 center Ovis family (Green) shows a stronger positive correlation, meaning performance improves as the model size increases. The **higher slope value** suggests that size contributes significantly to performance, but the lower slope indicates diminishing returns for larger models. From Fig. 6 Qwen2 family (Blue) displays a consistent scaling trend, with performance steadily increasing as model size grows. A **slope value (0.219)** suggests a steady performance gain, making Qwen2 models scalable and efficient.

Furthermore, whereas larger models generally perform better, gains become less significant beyond 40B+ parameters, especially in InternVL2 and Ovis families. The Qwen2 family exhibits a stable and predictable performance improvement, reinforcing its architectural optimizations and diverse training data. InternVL2 models show high variability, performance is inconsistent, implying that factors beyond size (e.g., dataset quality, pretraining strategy) impact results. Optimization is needed to enhance stability.

#### Implications for model selection

• **Small- to mid-sized models (10B–40B parameters)**: These models provide an optimal trade-off between performance and computational efficiency. Ovis and Qwen2 models perform best in this range.
• **Large models (>60B parameters)**: Despite their superior accuracy, they require significant computational resources and offer diminishing returns.
• **InternVL2 models require further optimization:** The variance in performance suggests that model improvements should focus on pretraining data and architectural refinements.

### Vision, text, and data

In this section, we present a summary of the selected VLMs, detailing their **language** and **vision** components, **parameter counts**, **data sizes**, and **data types**. This information is compiled from publicly available sources to offer insights into the underlying architectures and training data of these models. For a comprehensive breakdown, including detailed specifications, please refer to the **Supplementary material** (Section S2 and Table S2).

Key observations are:

• **General domain models:** Most of the selected models are trained on large-scale, general-purpose datasets that incorporate both text and vision modalities. For instance, models such as Qwen2-VL and LLaVA-NeXT leverage extensive general-domain data but are not specialized in medical applications.
• **Performance factors:** Vision models such as ViT-G/16 and CLIP ViT-L/14 are commonly integrated across various architectures, highlighting their robust feature extraction capabilities. The InternVL family employs InternViT architectures, designed to generate highly contextualized visual embeddings for general-purpose tasks.
• **Data size gap:** A notable limitation is the lack of publicly available information regarding dataset sizes for several general-purpose models. This raises concerns about benchmarking transparency and highlights the need for more structured dataset documentation.

## Integrating VLMs into clinical digital pathology workflow

Recent efforts in digital pathology, such as those by Cannizzaro Hospital^22^ and IPATIMUP,^21^ demonstrate that full digitization of histopathology workflows, when coupled with laboratory information system integration and standardized processes, can enable seamless AI adoption. These implementations highlight the growing need for model validation frameworks in clinical settings. Furthermore, benchmarks like PathMMU^52^ have shown that even state-of-the-art VLMs, such as GPT-4V, currently achieve only 49.8% accuracy on pathology-specific tasks, compared to 71.8% by human pathologists, underscoring the need for reliable evaluation frameworks like PathVLM-Eval before clinical deployment. PathVLM-Eval can be positioned upstream (for model selection and robustness testing). In the downstream, once a VLM is approved and deployed in the clinical workflow. It can be used to assist pathologists in case reporting by checking report quality, triage, and automating report generation.

Building upon these insights, as VLMs are increasingly capable of generating diagnostic reports.^25^^,^^70^ Our proposed work positions VLMs within both upstream and downstream stages of pathological operations. In a high-throughput lab receiving large number of whole slide images, in a results VLMs can saving time of pathologists, VLMs can screen all *N* cases and prioritize a smaller subset *K* (where K≪N) based on diagnostic relevance and urgency. These *K* cases are given higher priority by pathologists, who can edit or finalize draft reports, checking for consistency, completeness, and possible discrepancies. This closed-loop system helps reduce diagnostic delays, errors, improves triage accuracy, and standardizes reporting. Fig. 7 illustrates the integration of VLMs in clinical digital pathology.

## Conclusion and future recommendations

In this work, we present the most extensive benchmarking of open-source VLMs on the PathMMU dataset, evaluating over 60 state-of-the-art models across diverse histopathology-related tasks. Our study spans prominent model families, including LLaVA, Qwen-VL, Qwen2-VL, InternVL, Phi3, Llama3, MOLMO, and XComposer, providing the most comprehensive comparison to date in this domain. Our findings reveal key trade-offs between model size, multimodal integration, and domain-specific performance. Whereas larger models generally achieve higher accuracy, architecture optimizations, and fine-tuning strategies enable smaller models to remain competitive. Among all tested models, Qwen2-VL-72B-Instruct emerged as the top performer, achieving a remarkable average score of 63.97%, surpassing all other models across PathMMU subsets.This benchmark sets a new standard for evaluating VLMs in histopathology, providing a foundation for future advancements in medical AI.

Furthermore, based on these findings, we recommend that future evaluations of VLMs in histopathology include a systematic analysis of the dependency on visual input. Understanding which types of questions are more reliant on visual information can help in designing more robust models that leverage both visual and textual inputs effectively. Additionally, it would be beneficial to categorize questions based on their dependency on visual information. This would allow for a more granular analysis of model performance and help identify areas where visual input provides the most value. For example, categorizing questions into “text-driven,” “visual-driven,” and “hybrid” could provide deeper insights into the strengths and weaknesses of VLMs in pathology-related tasks.

In conclusion, whereas some questions can be answered accurately without visual input, there is still a clear benefit to providing visual information, especially for tasks that require detailed image analysis. The balance between visual and textual inputs should be carefully considered when developing and evaluating VLMs for medical applications.

## Declaration of competing interest

The authors declare that they have no known competing financial interests or personal relationships that could have appeared to influence the work reported in this article.

## Appendix A Supplementary data

Supplementary Material for: PathVLM-Eval: Evaluation of Open Vision Language Models in Histopathology Supplementary material includes: (S.1) Full benchmarking results of 60+ Vision-Language Models on the PathMMU subsets (PubMed, SocialPath, EduContent), and (S.2) Model specifications detailing architecture type, parameter size, vision encoders, and dataset characteristics. Supplementary material

## Footnotes

1 https://huggingface.co/allenai/MolmoE-1B-0924, accessed 1.Jan.2025.

2 https://huggingface.co/Cylingo/Xinyuan-VL-2B, accessed 1.Jan.2025.

3 https://huggingface.co/allenai/MolmoE-1B-0924, accessed 1.Jan.2025.

4 https://huggingface.co/meta-llama/Llama-3.2-11B-Vision-Instruct, accessed 1.Jan.2025.

## References

[1] Abdin M., Aneja J., Awadalla H. (2024). Phi-3 technical report: A highly capable language model locally on your phone. https://arxiv.org/abs/2404.14219.

[2] Achiam J., Adler S., Agarwal S. (2023). GPT-4 technical report. https://arxiv.org/abs/2303.08774.

[3] Alayrac J.B., Donahue J., Luc P. (2022). Flamingo: a visual language model for few-shot learning. Adv. Neural Inform. Process. Syst..

[4] Alexey D. (2020). An image is worth 16x16 words: transformers for image recognition at scale. https://arxiv.org/pdf/2010.11929/1000.

[5] Bai J., Bai S., Yang S. (2023). Qwen-vl: a frontier large vision-language model with versatile abilities. https://arxiv.org/abs/2308.12966.

[6] Ben Abacha A., Sarrouti M., Demner-Fushman D., Hasan S.A., Müller H. (2021). Proceedings of the CLEF 2021 Conference and Labs of the Evaluation Forum-Working Notes, 21-24 September 2021.

[7] Brown T., Mann B., Ryder N. (2020). Language models are few-shot learners. Advances in neural information process-. ing systems.

[8] Cai R., Song Z., Guan D. (2025). European Conference on Computer Vision.

[9] Chen L., Li J., Dong X. (2025). European Conference on Computer Vision.

[10] Chen Z., Wang W., Cao Y. (2024). Expanding performance boundaries of open-source multimodal models with model, data, and test-time scaling. https://arxiv.org/abs/2412.05271.

[11] Chen Z., Wang W., Tian H. (2024). How far are we to gpt-4v? closing the gap to commercial multimodal models with open-source suites. Science China Information Sciences.

[12] Chen Z., Wu J., Wang W. (2024). Proceedings of the IEEE/CVF Conference on Computer Vision and Pattern Recognition.

[13] Chiang W.L., Li Z., Lin Z. (2023). Vicuna: An open-source chatbot impressing gpt-4 with 90%* chatgpt quality, March 2023. https://lmsys.org/blog/2023-03-30-vicuna3.

[14] Contributors X. (2023). Xtuner: A Toolkit for Efficiently Fine-Tuning Llm. https://github.com/InternLM/xtuner.

[15] Dai W., Li J., Li D. (2023). Instructblip: Towards general-purpose vision-language models with instruction tuning. https://arxiv.org/abs/2305.06500.

[16] Deitke M., Clark C., Lee S., Tripathi R., Yang Y., Park J.S., Salehi M., Muennighoff N., Lo K., Soldaini L. (2025).

[17] Devlin J., Chang M.W., Lee K., Toutanova K. (2019). Proceedings of the 2019 conference of the North American chapter of the association for computational linguistics: human language technolo- gies, volume 1.

[18] Dong X., Zhang P., Zang Y. (2024). Internlm-xcomposer2: mastering free-form text-image composition and comprehension in vision-language large model. https://arxiv.org/abs/2401.16420.

[19] Duan H., Yang J., Qiao Y. (2024). Proceedings of the 32nd ACM International Conference on Multimedia.

[20] Dubey A., Jauhri A., Pandey A. (2024). The llama 3 herd of models. https://arxiv.org/pdf/2407.21783.

[21] Eloy C., Vale J., Curado M. (2021). Digital pathology workflow implementation at ipatimup. Diagnostics.

[22] Fraggetta F., Garozzo S., Zannoni G.F., Pantanowitz L., Rossi E.D. (2017). Routine digital pathology workflow: the catania experience. J. Pathol. Inform..

[23] Gao Z., Chen Z., Cui E. (2024). Mini-internvl: a flexible-transfer pocket multi- modal model with 5% parameters and 90% performance. Visual Intel- ligence.

[24] Guo Y., Jiao F., Shen Z., Nie L., Kankanhalli M. (2024). Unk-vqa: A dataset and a probe into the abstention ability of multi-modal large models. IEEE Transactions on Pattern Analysis and Machine Intelli- gence.

[25] Hartsock I., Rasool G. (2024). Vision-language models for medical report generation and visual question answering: a review. Front. Artif. Intel..

[26] He X., Zhang Y., Mou L., Xing E.P., Xie P. (2020). CoRR abs/2003.10286.

[27] Huang M., Liu Y., Liang D., Jin L., Bai X. (2024).

[28] Lau J.J., Gayen S., Ben Abacha A., Demner-Fushman D. (2018). A dataset of clinically generated visual questions and answers about radiology images. Scient. Data.

[29] Li B., Ge Y., Ge Y. (2024). Proceedings of the IEEE/CVF Conference on Computer Vision and Pattern Recognition.

[30] Li B., Wang R., Wang G., Ge Y., Ge Y., Shan Y. (2023). Seed-Bench: Benchmarking Multimodal LLMs with Generative Comprehension. https://arxiv.org/abs/2307.16125.

[31] Li J., Li D., Savarese S., Hoi S. (2023). International Conference on Machine Learning.

[32] Li Z., Yang B., Liu Q. (2024). Proceedings of the IEEE/CVF Conference on Computer Vision and Pattern Recognition.

[33] Liu B., Zhan L.M., Xu L., Ma L., Yang Y., Wu X.M. (2021). 2021 IEEE 18th International Symposium on Biomedical Imaging (ISBI).

[34] Liu H., Li C., Li Y., Lee Y.J. (2024). Proceedings of the IEEE/CVF Conference on Computer Vision and Pattern Recognition.

[35] Liu H., Li C., Li Y. (2024). Llava-next: Improved Reasoning, OCR, and World Knowledge. https://llava-vl.github.io/blog/2024-01-30-llava-next/.

[36] Liu H., Li C., Li Y. (2024).

[37] Liu H., Li C., Wu Q., Lee Y.J. (2024). Visual instruction tuning. Adv. Neural Inform. Process. Syst..

[38] Liu Y., Duan H., Zhang Y. (2025). European Conference on Computer Vision.

[39] Liu Y., Li Z., Huang M. (2024). Ocrbench: on the hidden mystery of ocr in large multimodal models. Sci. China Inform. Sci..

[40] Liu Y., Yang B., Liu Q. (2024). Textmonkey: An OCR-Free Large Multimodal Model for Understanding Document. https://arxiv.org/abs/2403.04473.

[41] Ltd., D (2024). mmalaya. https://github.com/DataCanvasIO/MMAlaya.

[42] Lu P., Bansal H., Xia T. Mathvista: Evaluating Mathematical Reasoning of Foundation Models in Visual Contexts. https://arxiv.org/abs/2310.02255.

[43] Lu S., Li Y., Chen Q.G. (2024). Ovis: structural embedding alignment for multimodal large language model. https://arxiv.org/abs/2405.20797.

[44] Mathew M., Karatzas D., Jawahar C. (2021). Proceedings of the IEEE/CVF Winter Conference on Applications of Computer Vision.

[45] Nayak S., Jain K., Awal R. (2024). Benchmarking vision language models for cultural understanding. https://arxiv.org/abs/2407.10920.

[46] OpenAI, a. Openai: Gpt-4v(ision) system card. https://cdn.openai.com/papers/GPTV_System_Card.pdf.

[47] OpenAI, b. Openai: Introducing chatgpt. https://openai.com/index/chatgpt/.

[48] OpenAI R. (2023).

[49] Qi L., Lv S., Li H. (2022). Findings of the Association for Computational Linguistics.

[50] Radford A., Kim J.W., Hallacy C. (2021). International Conference on Machine Learning.

[51] Raffel C., Shazeer N., Roberts A. (2020). Exploring the limits of transfer learning with a unified text-to-text transformer. J. Mach. Learn. Res..

[52] Sun Y., Wu H., Zhu C. (2024). European Conference on Computer Vision.

[53] Team G, Anil R., Borgeaud S. (2025). Gemini: a family of highly capable multimodal models. https://arxiv.org/abs/2312.11805.

[54] Touvron H., Lavril T., Izacard G. (2023). Llama: open and efficient foundation language models. https://arxiv.org/abs/2302.13971.

[55] Vaswani A., Shazeer N., Parmar N. (2017). Attention is all you need. Advances in neural information processing systems 30.

[56] Wang P., Bai S., Tan S. (2024). Qwen2-vl: enhancing vision-language model’s perception of the world at any resolution. https://arxiv.org/abs/2409.12191.

[57] Wang P., Bai S., Tan S. (2024). Qwen2-vl: enhancing vision-language model’s perception of the world at any resolution. https://arxiv.org/abs/2409.12191.

[58] Xu G., Jin P., Hao L., Song Y., Sun L., Yuan L. (2024). Llava-o1: let vision language models reason step-by-step. https://arxiv.org/abs/2411.10440.

[59] Xu P., Shao W., Zhang K. (2024). Lvlm-ehub: A comprehensive evaluation bench- mark for large vision-language models. IEEE Transactions on Pattern Analysis and Machine Intelligence.

[60] Yin Z., Wang J., Cao J. (2024). Lamm: language-assisted multi-modal instruction-tuning dataset, framework, and benchmark. Adv. Neural Inform. Process. Syst..

[61] Yu W., Yang Z., Li L. (2023). Mm-vet: evaluating large multimodal models for integrated capabilities. https://arxiv.org/abs/2308.02490.

[62] Yue X., Ni Y., Zhang K. (2024). Proceedings of the IEEE/CVF Conference on Computer Vision and Pattern Recognition.

[63] Zhan L.M., Liu B., Fan L., Chen J., Wu X.M. (2020). Proceedings of the 28th ACM International Conference on Multimedia.

[64] Zhang K., Li B., Zhang P. (2024). Lmms-eval: reality check on the evaluation of large multimodal models. https://arxiv.org/abs/2407.12772.

[65] Zhang P., Dong X., Wang B. (2023). Internlm-xcomposer: a vision-language large model for advanced text-image comprehension and composition. https://arxiv.org/abs/2309.15112.

[66] Zhang P., Dong X., Zang Y. (2024). Internlm-xcomposer-2.5: a versatile large vision language model supporting long-contextual input and output. https://arxiv.org/abs/2407.03320.

[67] Zhang X., Wu C., Zhao Z. (2023). Pmc-vqa: visual instruction tuning for medical visual question answering. https://arxiv.org/abs/2305.10415.

[68] Zhang Y.F., Zhang H., Tian H. (2024). Mme-realworld: could your multimodal llm challenge high-resolution real-world scenarios that are difficult for humans?. https://arxiv.org/abs/2408.13257.

[69] Zhao W., Ren X., Hessel J., Cardie C., Choi Y., Deng Y. (2024). Wildchat: 1m chatgpt interaction logs in the wild. https://arxiv.org/abs/2405.01470.

[70] Zhong Z., Wang Y., Wu J. (2024).

[71] Zhu D., Chen J., Shen X., Li X., Elhoseiny M. et al. 2023. Minigpt-4: Enhancing vision-language understanding with advanced large language models. https://arxiv.org/abs/2304.10592.

