# Supplementary material for: PathVLM-Eval: Evaluation of open vision language models in histopathology
Source: J Pathol Inform. 2025 Jun 5;18:100455. doi: 10.1016/j.jpi.2025.100455 (PMC12276438; doi:10.1016/j.jpi.2025.100455)
Supplement: Supplementary file 1 — Supplementary Material for: PathVLM-Eval: Evaluation of Open Vision Language Models in Histopathology Supplementary material includes: (S.1) Full benchmarking results of 60+ Vision-Language Models on the PathMMU subsets (PubMed, SocialPath, EduContent), and (S.2) Model specifications detailing architecture type, parameter size, vision encoders, and dataset characteristics. [file mmc1.pdf]

Supplementary Material for "PathVLM-Eval: Evaluation of Open Vision Language Models in Pathology"

S.1. Pathology Benchmarking Results

This section contains the full results of our pathology benchmarking. In this study, we evaluated 60+ recent VLMs on the PathMMU dataset, which features diverse tasks within pathology (see in Table. S.1). This benchmark builds upon prior work by Sun et al. [52], significantly expanding the evaluation scope by incorporating extensive visual and multimodal conditions using VLMEvalKit [19].

Table S.1. Benchmarking using VLMs on Pathology: We benchmark more 60 models on PathMMU dataset using VLMEvalKit [19] framework and we compare the results with existing benchmarks from PathMMU [52] to assess performance across various datasets.

Model	Rank	Avg Score	PubMed		SocialPath		EduContent	
			Tiny (281)	All (3068)	Tiny (229)	All (1805)	Tiny (255)	All (1938)
**LLaVA Series**								
LLaVA-Next-yi-34b	1	49.31	54.09	45.31	51.97	44.93	56.08	43.50
LLaVA-NeXT-Llama3	2	41.47	47.69	40.45	43.67	38.01	42.35	36.69
LLaVA-1.5-13B	3	39.26	45.90	37.32	39.73	37.45	39.60	35.60
LLaVA-NeXT-Mistral-7b	4	38.91	47.33	36.83	39.30	34.85	40.39	34.78
LLaVA-NeXT-Vicuna-13b	5	37.71	40.93	37.71	42.36	36.45	34.90	33.95
LLaVA-1.5-7B	6	37.38	43.77	39.6	36.34	38.01	31.37	35.34
LLaVA-NeXT-Vicuna-7b	7	37.19	43.06	36.34	39.30	34.63	35.29	34.52
LLaVA-Next-Interleave-7B-DPO	8	35.86	38.79	33.90	37.12	31.75	40.78	32.82
LLaVA-Next-interleave-7B	9	36.40	38.08	34.62	37.99	33.91	39.61	34.21
ShareGPT4v-7b	10	34.92	41.99	36.38	33.62	33.57	30.59	33.38
LLaVA-v1.5-13b-xtuner	11	34.99	37.37	36.31	36.68	32.80	34.12	32.71
ShareGPT4v-13b	12	34.10	40.57	35.04	34.06	31.47	32.55	30.96
LLaVA-Internlm-7b	13	32.65	33.81	34.94	33.19	28.59	35.29	30.13
LLaVA-Llama-3-8b	14	32.56	35.23	32.69	32.75	33.41	30.59	30.70
LLaVA-v1.5-7b-xtuner	15	31.36	34.52	33.18	33.19	29.58	29.41	28.33
**Qwen-VL Series**								
MiniMonkey	1	40.35	43.77	37.19	44.97	36.73	43.52	35.96
Monkey	2	35.02	41.64	35.39	31.88	33.30	34.11	33.84
Monkey-Chat	3	34.68	40.57	35.30	31.44	33.30	34.90	32.61
Qwen-VL-Chat	4	34.52	39.14	31.45	35.37	32.24	37.25	31.68
Qwen-VL-7B	5	32.45	32.38	32.26	30.56	31.91	36.07	31.52
**Qwen2-VL Series**								
Qwen2-VL-72B-Instruct	1	63.98	75.08	60.98	68.55	55.40	68.23	55.62
Qwen2-VL-7B-Instruct	2	55.35	62.63	54.791	57.20	49.86	56.86	50.77
XinYuan-VL-2B-Instruct	3	47.48	53.73	45.14	48.90	43.49	50.98	42.67
Qwen2-VL-2B-Instruct	4	45.53	48.75	44.58	49.78	41.88	45.49	41.43
**MOLMO Series**								
Molmo-7B-D-0924	1	42.31	47.33	41.32	43.23	41.55	41.17	39.31
Molmo-7B-O-0924	2	38.88	46.26	38.13	39.30	36.50	37.64	35.50
MolmoE-1B-0924	3	37.4	39.50	36.92	40.61	37.00	35.29	35.08
**Phi3 Series**								
Phi-3-Vision	1	39.04	44.83	38.55	38.86	38.83	36.07	37.10
Phi-3.5-Vision	2	43.77	49.11	44.16	44.97	40.55	43.92	39.93
**XComposer Series**								
XComposer2	1	37.74	40.56	36.60	36.68	35.34	41.96	35.34
Sharecaptioner	2	34.63	38.79	33.76	37.11	32.24	34.50	31.42
XComposer2_1.8b	3	31.73	35.94	32.65	30.13	31.63	30.58	29.46
**Intern-VL Series**								
InternVL-Chat-V1-2-Plus	1	52.36	58.36	50.39	51.96	48.08	56.86	48.55
InternVL-Chat-V1-2	2	51.09	58.71	47.75	55.02	46.14	54.50	44.42
InternVL-Chat-V1-1	3	34.98	37.36	34.97	34.06	34.62	36.86	32.04
**InternVL1.5 series**								
Mini-InternVL-Chat-4B-V1.5	1	52.34	55.52	50.74	57.20	46.65	55.69	48.24
Mini-InternVL-Chat-2B-V1.5	2	37.97	42.70	38.16	37.55	35.23	39.22	34.98
InternVL-Chat-V1-5	3	33.28	35.94	34.15	34.06	32.79	31.76	31.00
**InternVL2 series**								
InternVL2-40B	1	56.26	64.05	52.73	59.38	48.91	64.31	48.19
InternVL2-8B	2	49.9	58.01	45.33	57.64	43.99	51.76	42.67
InternVL2-4B	3	49.23	57.65	45.47	55.46	42.94	50.20	43.70
InternVL2-26B	4	47.61	53.02	44.69	53.28	41.72	50.20	42.77
InternVL2-1B	5	37.80	42.35	36.15	37.55	37.83	37.64	35.29
InternVL2-2B	6	39.57	41.99	37.45	43.23	36.18	42.35	36.22
**InternVL2 MPO series**								
InternVL2-8B-MPO	1	50.43	55.52	48.04	57.64	45.59	52.16	43.65
**InternVL2.5 series**								
InternVL2.5-38B	1	62.72	65.83	61.40	62.88	57.28	69.41	59.54
InternVL2.5-78B	2	62.49	71.17	57.33	67.24	54.62	71.76	52.83

Table S.1: continue

Model	Rank	Avg Score	PubMed Tiny (281)	PubMed All (3068)	SocialPath Tiny (229)	SocialPath All (1805)	EduContent Tiny (255)	EduContent All (1938)
**InternVL2.5 series**								
InternVL2.5-26B	3	54.40	60.14	52.44	61.57	46.98	57.64	47.67
InternVL2.5-8B	4	54.39	60.85	50.03	60.26	48.25	60.78	46.18
InternVL2.5-4B	5	53.23	62.98	49.60	54.14	46.26	59.60	46.85
InternVL2.5-2B	6	45.77	52.31	42.47	50.21	41.82	47.05	40.76
InternVL2.5-1B	7	42.19	49.46	38.95	46.72	39.44	40.00	38.59
**MMAlaya Series**								
MMAlaya	1	29.92	29.18	28.74	34.06	30.24	29.80	27.50
**Llama3 Series**								
Llama-3.2-11B-Vision-Instruct	1	24.07	23.48	23.85	24.45	22.32	27.45	22.91
**Ovis Series**								
Ovis1.6-Gemma2-27B	1	54.74	61.92	49.93	57.20	50.41	58.43	50.56
Ovis1.5-Llama3-8B	2	52.98	61.56	48.72	59.38	49.36	52.94	45.97
Ovis1.5-Gemma2-9B	3	52.45	59.07	50.52	55.02	48.25	55.29	46.59
Ovis1.6-Gemma2-9B	4	52.42	57.65	49.15	55.89	47.42	58.03	46.43

S.2. Vision, Text, and Data Information for Selected VLMs

The comprehensive overview of the selected multimodal models is presented in Table S.2, highlighting their language models, vision backbones, parameter scales, data sizes, and types of pretraining data. This comparison provides insights into the relative capabilities of various models and their suitability for multimodal understanding tasks.

Key highlights of the selected models:

- **Language Backbones**: The models utilize a diverse range of language models, including Vicuna, Llama, Qwen, Phi, and InternLM.
- **Vision Components**: Most models integrate CLIP ViT variants (e.g., ViT-L/14, ViT-G/16) or InternViT architectures for visual representation learning.
- **Scalability**: Parameter sizes vary significantly, from 2B (Qwen2-VL-2B-Instruct) to 78B (InternVL2.5-78B), demonstrating diverse modeling strategies for multimodal AI.

Table S.2. Overview of Selected VLM: Language and vision components, parameter counts, data sizes, and data types.

Model	Language Model	Vision Model	Params (B)	Data Size (Text/Vision)	Data Type
**LLaVA-v1.5-7B**	Vicuna-v1.5-7B	CLIP ViT-/14	7.2	(150k/595k)	General
**LLaVA-1.5-13B**	Vicuna-v1.5-7B	CLIP ViT-/14	13.4	(150k/595k)	General
**LLaVA-NeXT-Vicuna-7B**	Vicuna-v1.5-7B	CLIP ViT-L/14	7.1	1,318K	General
**LLaVA-NeXT-Vicuna-13B**	Vicuna-v1.5-13B	CLIP ViT-L/14	13.4	-	General
**LLaVA-NeXT-Mistral-7B**	Mistral-7B	CLIP ViT-L/14	7.6	1,318K	General
**LLaVA-NeXT-Llama3**	Llama-3-8B-Instruct	CLIP ViT-L/14	8	-	General
**Qwen-VL-7B**	Qwen-7B	ViT-G/16	9.6	-	General
**Qwen-VL-Chat**	Qwen-7B	ViT-G/16	9.6	(800/300)	General
**Qwen2-VL-7B-Instruct**	Qwen7-7B	ViT-600M	7	220k	General
**Qwen2-VL-2B-Instruct**	Qwen2-7B	ViT-600M	2	220k	General
**Qwen2-VL-72B-Instruct**	Qwen2-72B	QwenViT	73.4	220k	General
**MolmoE-1B-0924**	OLMoE-1B-7B-0924	CLIP ViT-L/14	7.2	17.4T	General
**Molmo-7B-D-0924**	Qwen2-7B	CLIP ViT-L/14	8	1M	General
**Molmo-7B-O-0924**	Qwen2-7B	CLIP ViT-L/14	8	1M	General
**Phi-3-Vision**	Phi-3	CLIP ViT-L/14	4.2	500B	General
**Phi-3.5-Vision**	Phi-3.5	CLIP ViT-L/14	4	500B	General
**Mini-InternVL-Chat-2B-V1.5**	InternLM2-1.8B	InternViT-300M	2	-	General
**InternVL2-4B**	Phi-3	InternViT-300M	4	-	General
**InternVL2-8B**	InternLM2.5-7B	InternViT-300M	8	-	General
**InternVL-Chat-V1-2**	InternViT-6B-448px-V1-2	Nous-Hermes-2-Yi-34B	40	1.2M	General
**InternVL2-40B**	Nous-Hermes-2-Yi-34B	InternViT-6B	40	-	General
**InternVL2-8B-MPO**	InternLM2.5-7B	InternViT-300M	8	-	General
**InternVL2.5-4B**	Qwen-2.5-3B	InternViT-300M-v2.5	4	-	General
**InternVL2.5-38B**	Qwen-2.5-32B	InternViT-6B-v2.5	38	-	General
**InternVL2.5-78B**	Qwen-2.5-72B	InternViT-6B-v2.5	78	-	General
**Llama-3.2-11B-Vision-Instruct**	Llama-3.1-8B	-	11	6B	General
**Ovis1.5-Llama3-8B**	Llama-3-8B-Instruct	SigLIP-400M	8	-	General
**Ovis1.6-Gemma2-27B**	Gemma2-27B	SigLIP-400M	28.9		
